# Characterization of the GHB Withdrawal Syndrome

**DOI:** 10.3390/jcm10112333

**Published:** 2021-05-26

**Authors:** Casper J. H. Wolf, Harmen Beurmanjer, Boukje A. G. Dijkstra, Alexander C. Geerlings, Marcia Spoelder, Judith R. Homberg, Arnt F. A. Schellekens

**Affiliations:** 1 Medical Center, Department of Psychiatry, Radboud University, 6525 GA Nijmegen, The Netherlands; alexandergeerlings@hotmail.com (A.C.G.); Arnt.Schellekens@radboudumc.nl (A.F.A.S.); 2 Department of Cognitive Neuroscience, Donders Institute for Brain Cognition and Behaviour, Radboud University, 6525 EN, Nijmegen, The Netherlands; marcia.spoelder-merkens@radboudumc.nl (M.S.); Judith.Homberg@radboudumc.nl (J.R.H.); 3 Nijmegen Institute for Scientist-Practitioners in Addiction (NISPA), 6525 HR Nijmegen, The Netherlands; harmen.beurmanjer@novadic-kentron.nl (H.B.); boukje.dijkstra@outlook.com (B.A.G.D.); 4 Novadic-Kentron Addiction Care, 5261 LX Vught, The Netherlands

**Keywords:** gamma-hydroxybutyric acid, GHB, addiction, withdrawal, dependence, detoxification, abstinence, treatment, sex differences

## Abstract

The gamma-hydroxybutyric acid (GHB) withdrawal syndrome can have a fulminant course, complicated by severe complications such as delirium or seizures. Detoxification by tapering with pharmaceutical GHB is a safe way to manage GHB withdrawal. However, a detailed description of the course of the GHB withdrawal syndrome is currently lacking. This study aimed to (1) describe the course of GHB withdrawal symptoms over time, (2) assess the association between vital signs and withdrawal symptoms, and (3) explore sex differences in GHB withdrawal. In this observational multicenter study, patients with GHB use disorder (*n* = 285) were tapered off with pharmaceutical GHB. The most reported subjective withdrawal symptoms (SWS) were related to cravings, fatigue, insomnia, sweating and feeling gloomy. The most prevalent objective withdrawal symptoms (OWS) were related to cravings, fatigue, tremors, sweating, and sudden cold/warm feelings. No association between vital signs and SWS/OWS was found. Sex differences were observed in the severity and prevalence of specific withdrawal symptoms. Our results suggest that the GHB withdrawal syndrome under pharmaceutical GHB tapering does not strongly differ from withdrawal syndromes of other sedative drugs. The lack of association between vital signs and other withdrawal symptoms, and the relative stability of vitals over time suggest that vitals are not suitable for withdrawal monitoring. The reported sex differences highlight the importance of a personalized approach in GHB detoxification.

## 1. Introduction

The repeated use of the recreational drug γ-hydroxybutyric acid (GHB) can lead to GHB use disorder (GUD) [1,2]. Similar to other substance use disorders (SUDs), GUD is characterized by a loss of control over GHB intake and physical dependence on GHB [3]. In 2019, the prevalence of GHB use in European countries varied from 0.1% in adults (16–64 year old) to 1.7% among young adults (16–34 year old) [2]. Although GUD has a relatively low prevalence compared to other SUDs, its societal and financial impact are disproportionally high. GHB use is involved in ~12% of drug-related emergency care cases in Europe, caused by accidental overdosing or severe withdrawal symptoms upon sudden abstinence [2,4].

Due to the rapid onset of action (Tmax = 25–40 min) and the short half-life (T½ = 30–60 min) of GHB, patients with GUD typically consume GHB every 2–3 h to prevent withdrawal symptoms [5,6]. Cessation of GHB use results in a severe withdrawal syndrome, characterized by an erratic and fulminant course. Reported symptoms of GHB withdrawal are tremors, agitation, anxiety, hallucinations, psychoses and delirium [6,7]. Abrupt GHB withdrawal without adequate treatment leads to delirium in over 50% of cases [7].

Treatment of GHB withdrawal during detoxification aims to reduce the severity of withdrawal symptoms. Two commonly used methods for GHB detoxification are benzodiazepine tapering and the more extensively studied pharmaceutical GHB tapering. Benzodiazepine administration increases GABAergic signaling through the GABAA receptor, and requires dose regimens of up to six times per day [8,9]. In contrast, pharmaceutical GHB activates both the GABAB and GHB receptor, and is administered to the patient every two to three hours [10].

Some studies suggest that pharmaceutical GHB tapering is a safer method for detoxification compared to benzodiazepines [6,8]. This might be related to the complex pharmacological profile of GHB. Studies have shown that low doses of GHB primarily affect the metabotropic GHB receptor, causing an increase in glutamatergic signaling and a decrease in GABAergic signaling [11,12]. In contrast, high doses of GHB also activate the GABAB receptor, decreasing glutamatergic signaling and increasing GABAergic signaling [13,14]. Benzodiazepines, acting at GABAA receptors, might therefore not sufficiently suppress GABAB-mediated GHB withdrawal, leading to an increased risk for adverse events during benzodiazepine detoxification, such as delirium [8].

Despite the existing evidence for the safety of pharmaceutical GHB tapering, several important issues regarding this approach remain to be elucidated [6,15]. For instance, little is known about the development of individual withdrawal symptoms over time during GHB detoxification. Additionally, some suggest to base the speed of tapering on the monitoring of vital signs, such as blood pressure and heart rate [16,17,18], whereas others propagate the monitoring of (subjective) withdrawal symptoms [15,19]. Understanding the development of individual withdrawal symptoms over time, and their relationship with vital signs, could facilitate the effective dosing and monitoring of the detoxification process.

The GUD population is characterized by a substantial proportion of women of about one third [6,20]. Women with SUDs are known to show higher rates of internalizing psychiatric symptoms, such as depression and anxiety, whereas men with SUDs show higher rates of externalizing symptoms, such as antisocial personality [21,22,23]. It has been shown that women with GUD experience stronger withdrawal compared to men with GUD [6]. However, detailed information on the exact nature of these sex differences is lacking.

The aim of the current study is to further our understanding of the GHB withdrawal syndrome, in order to improve medical treatment of this condition. We characterize the course of the GHB withdrawal syndrome during inpatient pharmaceutical GHB detoxification in a large database of clinical observations in patients with GUD. Specifically, we analyze: 1. the course of individual withdrawal symptoms over time, 2. the association between vital signs and subjective withdrawal symptoms, and 3. sex differences in the course of the GHB withdrawal syndrome.

## 2. Materials and Methods

### 2.1. Study Design

We used data from two large observational multicenter studies in patients with GUD. The main focus of these studies was to assess the safety of detoxification with pharmaceutical GHB, as published elsewhere [6,24]. Both studies had similar inclusion criteria, treatment paradigms, and outcome measures. The Medical Ethical Research Committee Twente and Central Committee on Research Involving Human Subjects approved the study protocols and considered that the study did not fall under the scope of the Medical Research Involving Human Subjects Act (WMO). The off-label use of pharmaceutical GHB for GHB detoxification was approved by the Dutch Health Care Inspection.

### 2.2. Participants

**Figure 1:**
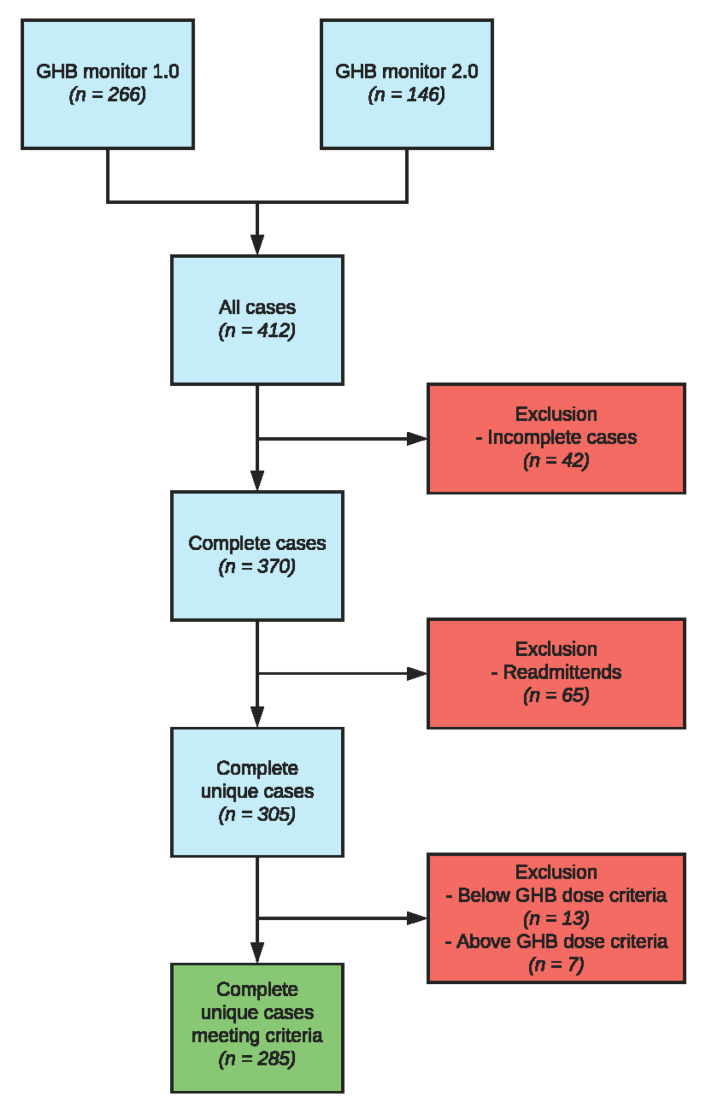
Flowchart of participants included in the study.

Inpatients being treated for GUD (*n* = 412) at one of the seven participating addiction treatment centers in the Netherlands (Novadic-Kentron, Tactus, IrisZorg, Victas, Verslavingszorg Noord-Nederland, Brijder and Mondriaan GGZ) were included between 2011 and 2015. Patients were between 18 and 60 years old. All patients were classified with GHB dependence according to the Diagnostic and Statistical Manual of Mental Disorders IV-TR [25] general criteria for psychoactive substance dependence. Patients were excluded from the study if they could not speak or read the Dutch language, if they suffered from a severe co-morbid psychiatric condition that required immediate attention (e.g., psychosis, manic episodes, or suicidal ideation), or in the case of pregnancy [6]. Patients were excluded from data analyses if they had less than three tapering days, or if their GHB dose before admission was below 30 milliliters (since these patients should have been treated ambulatory) or above 240 milliliters (since these patients showed an aberrant, non-representative GHB withdrawal syndrome under pharmaceutical GHB tapering). The threshold of >240 mL was determined by adding 2.5 SDs to the daily average consumption volume. If patients were included in both monitors, data of the first treatment episode were used. This resulted in a database of 285 complete, unique patients with GUD undergoing inpatient GHB detoxification (Figure 1).

### 2.3. Instruments

Demographics and other clinical data were obtained from chart reviews (admission data, discharge data and the discharge summary). Measurements in the Addictions for Triage and Evaluation (MATE) Section 1 was used to assess current substance use (past 30 days), lifetime substance use, and the classification of substance dependence according to DSM-IV [26]. In Dutch addiction treatment centers, the MATE is the standard clinical assessment tool, and has proven to have good psychometric quality [26]. The GHB questionnaire, specifically assessing the pattern of previous GHB use, was used in addition to the MATE [6]. The GHB questionnaire consists of 23 parameters, including total years of use, daily dose, volume per dose and time interval between doses. The questionnaire is commonly used in Dutch addiction treatment centers that treat patients with GUD.

#### 2.3.1. Subjective Withdrawal Scale

The subjective withdrawal scale consists of 33 items representing individual withdrawal symptoms. Patients indicate to what degree they experience each symptom on a 5-point Likert scale (0 = not at all, 1 = a little, 2 = moderately, 3 = quite a bit, 4 = extremely). The subjective withdrawal scale is based on the format of the Subjective Opiate Withdrawal Scale [27], extended with subjective withdrawal symptoms of other psychoactive substances as described in the DSM-IV-TR [25]. Its Dutch translation has good psychometric properties in opioid-dependent inpatients [28].

#### 2.3.2. Objective Withdrawal Scale

The objective withdrawal scale consists of 34 observable signs of withdrawal. It is composed of symptoms included in the Objective Opiate Withdrawal Scale [27] and objective withdrawal symptoms of other psychoactive substances as described in the DSM-IV-TR [25]. The objective withdrawal scale is filled in by health professionals (mostly nursing staff), where symptoms are classified as present (1) or absent (0). The objective and subjective withdrawal scale have been reported in several previous studies assessing GHB withdrawal [6,8], and are the standard GHB withdrawal assessment scales in addiction treatment centers in the Netherlands. As a result, clinical staff are experienced with applying these instruments in their daily routine. Furthermore, prior to the data collection of both samples, all nursing staff received instructions and training in how to handle the withdrawal scales.

#### 2.3.3. Vitals

Vital signs (heart rate, systolic- and diastolic blood pressure) were measured by the nursing staff. Vitals were annotated under the objective withdrawal scale.

### 2.4. Procedure

Upon admission to the addiction treatment center, information on GHB use and GUD was acquired by trained study nurses through the above-mentioned questionnaires. The detoxification procedure consisted of three phases: titration, tapering, and recovery. During the titration phase, patients were treated with pharmaceutical GHB that was 70% of the reported self-administered illicit GHB dose (based on an average ‘street’ concentration of 650 mg/mL). The GHB dose was increased in the case of withdrawal and decreased in the case of sedation, until the pharmaceutical GHB dose was found on which patients were stable and experienced neither withdrawal nor sedation. This usually took between one and two days, after which, the tapering phase started. During the tapering phase, the GHB dose was lowered by 300 mg of the GHB per dose per day. The interval between doses was usually two to three hours. Symptoms were assessed 30 min prior to each GHB dose. The tapering phase lasted 11 days on average. The recovery phase started when the pharmaceutical GHB was tapered to 0, which lasted six days on average. For a more detailed description of the protocol, see Dijkstra et al. (2017) [6].

### 2.5. Data Analysis

Demographics were summarized using descriptive statistics and compared between men and women using one-way MANOVAs (GHB use characteristics (including age, age at first GHB use, mean years of GHB use, mean days of GHB use, mean daily GHB dose, mean interval between two GHB doses) and co-morbid substance use) and Pearson chi-square test (medication).

To describe the general course of GHB withdrawal, we examined the first 11 days of tapering, since the average tapering period lasted 10.3 days. Linear mixed model analysis was performed to assess the development of total SWS/OWS scores over time. Mean daily SWS/OWS scores were used as dependent variables.

We visualized symptom severity and prevalence using heat maps. The average relative symptom severity and prevalence were calculated by dividing the average symptom score on the respective scale by the maximum possible score on that scale. To examine the development of individual withdrawal symptoms over time, we performed descriptive statistics. Pearson correlation analysis was performed to assess the association of individual withdrawal symptoms between both scales. Bonferroni corrections were applied to correct for multiple comparisons.

Linear mixed model analysis was performed to assess the development of vital signs over time. The mean daily scores of the vital parameters were used as dependent variables. Pearson correlation analysis was performed to assess the association of vital signs with daily average SWS/OWS scores. Bonferroni corrections were applied to correct for multiple comparisons.

Finally, to explore sex differences in the course of withdrawal symptoms over time, we performed linear mixed model analysis. Daily average SWS and OWS scores were used as the dependent variables, and sex was used as an independent variable. Sex differences in the severity and prevalence of individual withdrawal symptoms were analyzed using one-way MANOVA, using average scores per patient per symptom across the entire tapering period.

ANOVAs, chi-square tests, correlations, heat map analyses and linear mixed model analyses were carried out with the Statistical Package for the Social Sciences (SPSS) (25.0) and with GraphPad Prism (9.0). Significance was set at *p* < 0.05.

## 3. Results

### 3.1. Demographics

Demographic characteristics of participants (*n* = 285) are presented in Table 1. Men and women differed in GHB-related characteristics (Table 1: one-way MANOVA, F_(6,174)_ = 2.227, *p* < 0.05; Wilk’s λ = 0.929). Men included in the analysis were older and started using GHB at a later age compared to women (Table 1: age (F_(1,179)_ = 8.978, *p* < 0.01); age at first GHB use (F_(1,179)_ = 6.797, *p* < 0.01)). Men and women did not differ in rates of co-morbid substance use or in the prevalence of medication use.

### 3.2. Development of Withdrawal Symptoms over Time during GHB Detoxification

**Figure 2:**
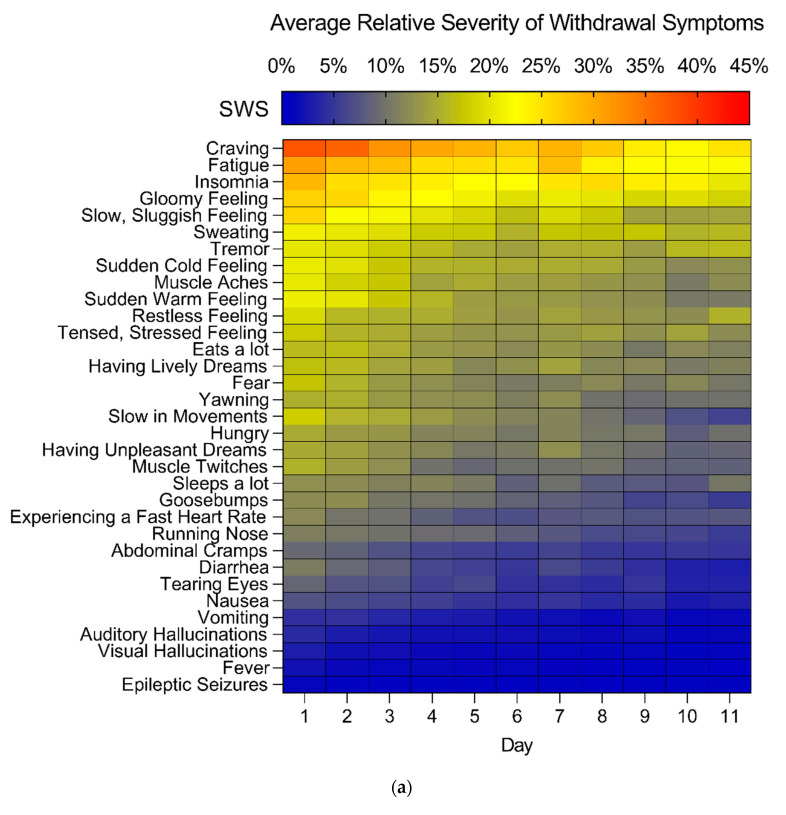

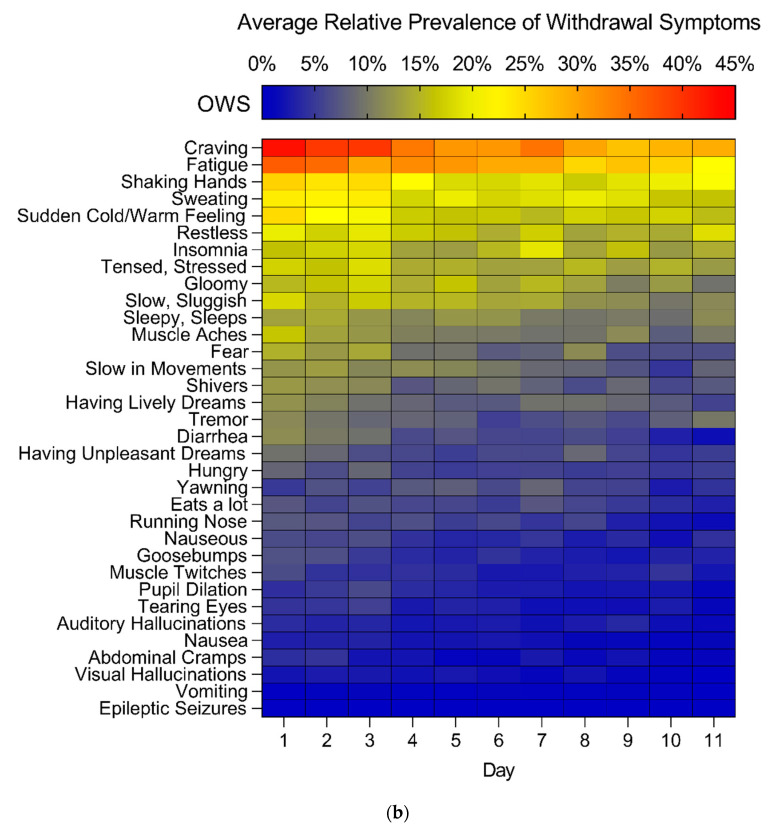
Average relative severity and prevalence of subjective- and objective withdrawal symptoms. (**a**) Heat map of all 33 SWS over time for the first 11 detoxification days. Symptoms are ranked based on the average severity of the symptom over all days for males and females combined. (**b**) Heat map of all 34 OWS over time for the first 11 detoxification days. Symptoms are ranked based on the average presence of the symptom over all days for males and females combined.

The total SWS and OWS scores gradually decreased across the tapering phase (linear mixed models, SWS: main effect of time, F_(10,198)_ = 12.185, *p* < 0.0001; OWS, main effect of time, F_(10,209)_ = 9.639, *p* < 0.0001). The most severely experienced SWS were “craving”, “fatigue”, “insomnia” and a “gloomy” and “slow, sluggish feeling” (Figure 2). The most often reported OWS by nurses were “craving” and “fatigue”, next to the symptoms “shaking hands”, “sweating” and a “sudden cold/warm feeling” (Figure 2).

The SWS “muscle aches”, “muscle twitches”, “tensed, stressed feeling”, “experiencing a fast heart rate” and “abdominal cramps” were most severe in the first part of detoxification (first three days). Over the first four days, a >70% decrease in severity was reported for these symptoms. In contrast, several SWS (“sweating”, “tremor”, “sleeps a lot”, and “restless feeling”) remained stable over time (<25% decrease in severity over 11 days). On average, there were no SWS that became more severe during the tapering phase.

The OWS “sweating”, “sudden cold/warm feeling”, “muscle aches”, “tensed, stressed feeling”, “shivers”, “having unpleasant dreams”, “hungry”, and “goosebumps” were primarily present in the first part of detoxification (first 3 days). Over the first four days, a >70% decrease in prevalence was observed for these symptoms. The OWS “tremor”, “shaking hands”, “sleepy, sleeps”, “insomnia”, “restless” and “yawning” were consistently present over time (<25% decrease in presence over 11 days). The OWS “yawning”, “insomnia”, “gloomy” and “visual hallucinations” were on some days more prevalent compared to day 1. The majority of SWS and OWS that appeared on both scales showed a moderate to strong correlation with the corresponding symptom on the other scale (r = 0.226 to 0.826, *p* < 0.0015 after Bonferroni correction) (Table A1).

**Figure 3:**
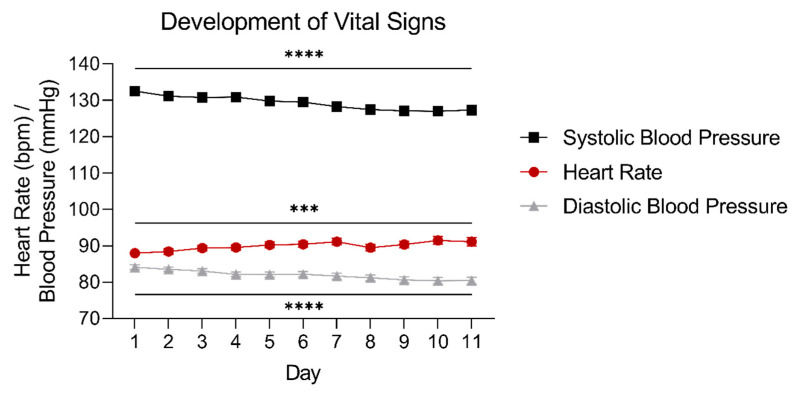
Development of vital signs over time during the first 11 days of GHB tapering. Data are presented as the average of all patients measured on the respective days ± SEM (*n* = 108–263). Error bars may lie under the symbol of the graph. *** = main effect of time *p* < 0.001; **** = main effect of time *p* < 0.0001.

The average heart rate gradually increased over time during detoxification from 87.9 to 91.1 bpm (linear mixed models, heart rate: main effect of time: F_(10,192)_ = 3.509, *p* < 0.001). The average systolic and diastolic blood pressure gradually decreased over time from 132.5 to 127.3 mmHg, and from 84.1 to 80.4 mmHg, respectively (linear mixed models, systolic blood pressure main effect of time: F_(10,196)_ = 5.848, *p* < 0.0001; diastolic blood pressure main effect of time: F_(10,183)_ = 10.095, *p* < 0.0001), as shown in Figure 3.

### 3.3. Association between Vital Signs and Subjective- and Objective Symptoms of GHB Withdrawal

Overall, no correlations were observed between vital signs and daily average SWS scores (Table A2) or between vital signs and daily average OWS scores after correction for multiple testing (*p* > 0.0015), see Table A3.

### 3.4. Differences in GHB Withdrawal Syndrome between Men and Women

**Figure 4:**
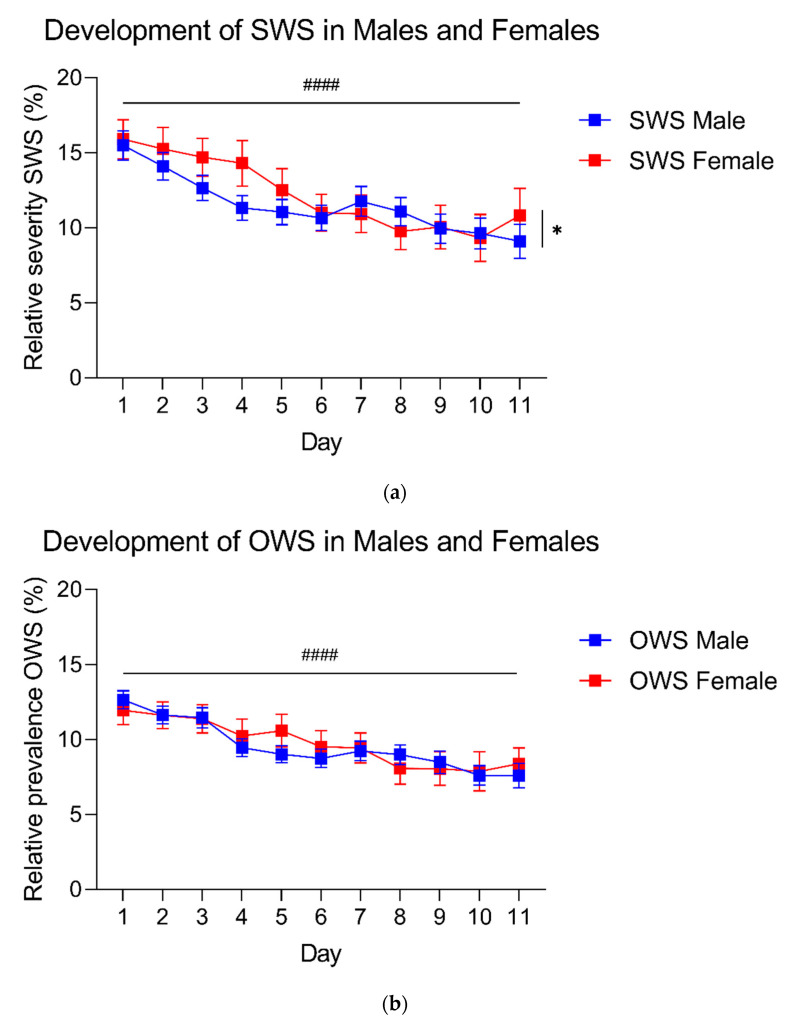
Development of subjective- and objective withdrawal symptoms over time in males and females. (**a**) Development of subjective withdrawal symptoms (SWS) during GHB detoxification, divided by sex. SWS are presented as the average severity of all 33 measured SWS during the day (males *n* = 74–184, females *n* = 31–76). (**b**) Development of objective withdrawal symptoms (OWS) during GHB detoxification, divided by sex. OWS are presented as the average presence of all 34 measured objective withdrawal symptoms during the day (males *n* = 77–191, females *n* = 31–72). Data are presented as mean ± SEM. #### = main effect of time *p* < 0.0001; * = time x sex interaction *p* < 0.05.

In contrast to what we expected, there was no difference in total SWS and OWS scores between men and women across the tapering phase (Figure 4: linear mixed models, no effect of sex), and both sexes showed a similar decrease in total OWS score over time (Figure 4: linear mixed models, OWS no significant interaction). Men and women showed a slightly different course of total SWS score over time (Figure 4: linear mixed models, SWS time x sex interaction, F_(10,198)_ = 2.038, *p* < 0.05).

One-way MANOVA showed that men and women differed in the severity of individual SWS (F_(33,233)_ = 2550, *p* < 0.001; Wilk’s λ = 0.735). Specifically, women scored higher on fear (F_(1,265)_ = 4.531, *p* < 0.05), gloomy feeling (F_(1,265)_ = 7.507, *p* < 0.01), yawning (F_(1,265)_ = 6.132, *p* < 0.05), goosebumps (F_(1,265)_ = 6.272, *p* < 0.05), sweating (F_(1,265)_ = 7.583, *p* < 0.01), tearing eyes (F_(1,265)_ = 6.863, *p* < 0.01), muscle aches (F_(1,265)_ = 9.357, *p* < 0.01), nausea (F_(1,265)_ = 4.700, *p* < 0.05), craving (F_(1,265)_ = 7.519, *p* < 0.01), sudden cold feelings (F_(1,265)_ = 13.248, *p* < 0.001), and sudden warm feelings (F_(1,265)_ = 7.979, *p* < 0.01), whereas men were more often reported to eat a lot during detoxification compared to women (F_(1,265)_ = 14.059, *p* < 0.001), see Figure 2).

**Figure A1:**
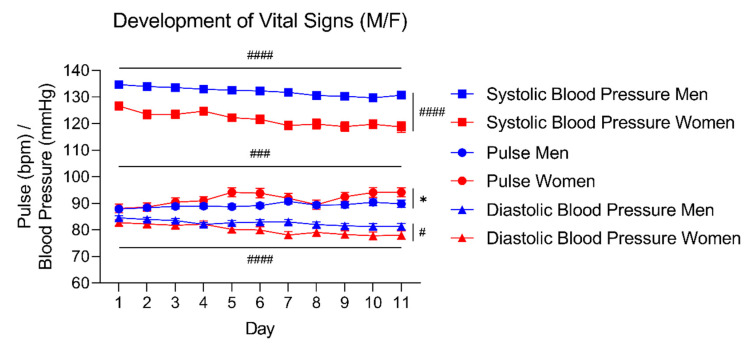
Development of vital signs over time in males and females during the first 11 days of GHB tapering. Data presented as mean ± SEM. Error bars are not shown if they are shorter than the size of the symbol. # = main effect of sex, *p* < 0.05; ### = main effect of time, *p* < 0.001; #### = main effect of time/sex, *p* < 0.0001; * = time × sex interaction, *p* < 0.05.

One-way MANOVA showed that men and women differed in prevalence of individual OWS (F_(34,234)_ = 2.004, *p* < 0.01; Wilk’s λ = 0.774). Women showed more shivering (F_(1,267)_ = 16.046, *p* < 0.0001), sudden cold/warm feelings (F_(1,267)_ = 5.664, *p* < 0.05, abdominal cramps (F_(1,267)_ = 6.665, *p* < 0.05), nausea (F_(1,267)_ = 10.103, *p* < 0.01) and vomiting (F_(1,267)_ = 4.492, *p* < 0.05), while men showed more insomnia (F_(1,267)_ = 5.024, *p* < 0.05) and eating a lot (F_(1,267)_ = 7.382, *p* < 0.01). Additionally, men showed a higher blood pressure than women (Figure A1).

## 4. Discussion

This study set out to analyze the course of the GHB withdrawal syndrome in patients with GUD during inpatient detoxification with pharmaceutical GHB. The GHB withdrawal syndrome was primarily characterized by sleep-related symptoms, mood-related symptoms and several physiological symptoms, including sweating and tremors. The majority of symptoms steadily declined in severity over time, while some symptoms (e.g., tremors, sleeping a lot) were not strongly affected by GHB tapering. Vital signs did not correlate with other withdrawal symptoms. Women showed a different pattern of withdrawal symptoms compared to men.

The most prominent withdrawal symptoms that decreased over time include “craving”, “fatigue”, “insomnia”, “gloomy”, “slow, sluggish”, “sudden cold/warm feeling”, “muscle aches” and “tensed, stressed”, and might represent the core symptoms of GHB withdrawal. Other withdrawal symptoms that were frequently present during detoxification include “sweating”, “tremor” and “restlessness”. Withdrawal syndromes of other sedatives, such as alcohol and benzodiazepine withdrawal, show overlap with symptoms seen in this study, such as anxiety/fear, tremor, sweating, insomnia (alcohol/benzodiazepines), restlessness and muscle twitches (benzodiazepines) [29,30]. Other characteristic alcohol withdrawal symptoms such as hypertension, tachycardia, and fever were hardly seen in our sample [31,32]. Similarly, severe GHB withdrawal symptoms such as epileptic seizures, hallucinations and delirium, were rare in our sample, probably because GHB tapering dampened the overall severity of withdrawal symptoms. Future studies should address whether other withdrawal scales, for instance the Clinical Institute Withdrawal Assessment for Alcohol (CIWA-Ar) tool, can also reliably be used to guide tapering [33]. Indeed, the CIWA-Ar has also been used to assess GHB withdrawal in two case reports [34,35].

Several symptoms present at the start of detoxification were not strongly affected by GHB tapering (“sweating”, “tremor”, “sleeps a lot”, ”sleepy”, “sleeps”, “restless feeling”, ”restless”, “yawning” and “shaking hands”). This may reflect a more long-term dysregulation of autonomic processes, for instance, due to a chronically disrupted sleep pattern. Several of these symptoms were also still present upon discharge of GHB detoxification treatment, including “craving” and “insomnia”. The presence of several symptoms following detoxification, including sleep-related disturbances, might also contribute to the high relapse rates seen with GUD, which is also observed with other SUDs such as alcohol, cocaine and opioid use disorder [36]. Aftercare following detoxification should therefore aim at reducing these symptoms that persist after detoxification, such as sleep-related issues and cravings.

Contrary to other substance withdrawal syndromes, we did not find an association between vital signs and objective or subjective GHB withdrawal symptoms [37,38]. In several other substance withdrawal syndromes, vitals are associated with withdrawal symptom severity and are therefore used as an indicator for overall withdrawal severity. For instance, in alcohol, vital signs are used to determine titration and tapering regimes during detoxification [3,38,39,40]. Our findings suggest that changes in vital signs during GHB detoxification may not be suitable for the monitoring of GHB withdrawal severity.

Additionally, both the increase in heart rate (from 87.9 to 91.1 bpm on average) and the decreases in systolic and diastolic blood pressure (from 132.5/84.1 to 127.3/80.4 mmHg on average) we observed here were rather small and of little clinical relevance, despite being statistically significant. Yet, with sudden GHB withdrawal, tachycardia and hypertension are often observed [7]. Our results indicate that GHB tapering might have prevented a derailment of vital signs, implicating that a change in withdrawal symptoms may precede a derailment of vital signs during GHB detoxification, as also suggested by Beurmanjer et al. (2020) [8].

Men and women showed different types of withdrawal symptoms. Specifically, women scored higher on average on a large variety of (mainly subjective) individual withdrawal symptoms. This is also observed with other SUDs such as opioids, cannabis and nicotine [41,42,43]. The differences in GHB withdrawal symptoms between males and females may be partially related to differences in co-occurring psychiatric conditions. Dijkstra et al. (2017) showed that patients with GUD with higher baseline levels of depression, anxiety and stress experienced higher levels of subjective withdrawal [6]. In addition, co-morbid mood and anxiety disorders are more common in women with SUDs compared to men with SUDs [21,22,23], possibly explaining the increased severity of several individual withdrawal symptoms in women during GHB detoxification compared to men. In contrast to the findings of Dijkstra et al. (2017), we did not find a difference in total SWS severity between men and women across the tapering phase [6]. This may be explained by the fact that Dijkstra et al. (2017) included the titration-, tapering- and recovery days, whereas we only focused on the tapering phase. The limited suppression of (co-morbid) symptoms during titration and recovery days might account for the observed differences. The reported differences between men and women suggest that women might benefit from more gradual tapering strategies compared to men.

In the assessment of GHB withdrawal severity, both objective and subjective symptoms were measured. There is a large overlap between the objective and subjective withdrawal scales regarding the type of symptoms assessed. It can be questioned whether both scales are required to obtain a complete picture of withdrawal severity. The current data show that subjective withdrawal severity generally parallels clinical observations by nursing staff, as also seen with, e.g., opioid withdrawal [28]. However, the subjective withdrawal scale seems more sensitive to a change in withdrawal severity, probably as a result of the 5-point Likert scale design compared to the dichotomous objective withdrawal scale. It might thus be sufficient to focus on self-reported withdrawal severity to monitor GHB detoxification.

The current findings should be viewed in light of several study limitations. A relatively high proportion of patients in our study showed co-morbid substance use (Table 1), possibly contributing to the observed withdrawal symptoms. However, the observed prevalence of co-morbid substance use is representative for the population of patients with GUD [44]. On the one hand, the high rates of co-morbid substance use hamper firm conclusions about the specific effects of GHB withdrawal. On the other hand, clinical reality is that people with GUD often have co-morbid SUDs, thus making our observations clinically relevant [44].

The withdrawal scales used in this study were originally based on opioid withdrawal scales [27], and complemented with other symptoms based on the DSM-IV [25]. Although a total of 38 individual withdrawal signs and symptoms were assessed, it is still possible that withdrawal symptoms that are unique for GHB withdrawal were not assessed with the current withdrawal scales. Throughout detoxification, patients repeatedly mentioned that they had an itchy feeling. This symptom may be considered to be included in the GHB withdrawal scale.

During GHB detoxification, pharmacological treatment for co-morbid psychiatric disorders, such as benzodiazepines, selective serotonin reuptake inhibitors (SSRIs) or anti-psychotics, continued. We speculate this may have dampened the severity of GHB withdrawal, possibly causing our results to be an underestimation of the severity of GHB withdrawal compared to when only pharmaceutical GHB is provided, as also suggested in previous reports [9,45]. The effects of other medications on the course of the GHB withdrawal syndrome requires further study.

It is also important to note that the current findings do not generalize to other methods for GHB detoxification, such as benzodiazepine tapering (different receptor systems), or acute unassisted GHB detoxification (cold turkey). Severe withdrawal symptoms, such as epileptic seizures and psychotic symptoms that were hardly observed here, might be more common in such cases [7,8].

## 5. Conclusions

The GHB withdrawal syndrome during pharmaceutical GHB tapering is characterized by a variety of symptoms that fade over time, and which are also commonly observed during alcohol and benzodiazepine withdrawal. The observed lack of association between vitals and subjective or objective withdrawal symptoms, and the limited variation of vitals over time, question their relevance as an indicator for GHB withdrawal severity during detoxification. Finally, women experience qualitatively different GHB withdrawal symptoms during GHB detoxification compared to men. Our research suggests that the subjective withdrawal scale may serve as a basis to personalize tapering speed in order to minimize withdrawal severity, and account for sex differences in the GHB withdrawal syndrome.

## Figures and Tables

**Table 1:** Patients with GUD characteristics of unique patients (*n* = 285).

Characteristics		Male (*n* = 206)	Female (*n* = 79)
Sex		72.3%	27.7%
Mean age in years (SD) **		29.34 (6.44)	26.63 (6.67)
Mean age at first GHB use (SD) **		25.05 (6.67)	21.96 (6.62)
Mean years of GHB use (SD)		4.18 (2.62)	3.83 (2.96)
Mean days of GHB use in last 30 days (SD)		29.69 (1.74)	29.91 (0.71)
Mean daily GHB dose before admission in mL (SD)		92.01 (48.75)	76.44 (43.05)
Mean interval between two GHB doses in hours (SD)		2.30 (5.88)	1.85 (0.64)
Mean number of days of co-morbid substance use in last 30 days (SD)	Alcohol	4.95 (8.87)	2.96 (5.77)
	Nicotine	20.97 (13.55)	23.04 (12.64)
	Cannabis	6.68 (11.56)	5.73 (10.60)
	Stimulants	5.46 (10.23)	5.35 (10.07)
	Cocaine	2.11 (5.97)	1.64 (0.95)
	Sedatives	8.16 (12.88)	8.87 (12.92)
Medication	Anti-psychotics	15.53%	12.66%
	Beta blockers	3.88%	2.53%
	Benzodiazepines	40.78%	41.77%
	Sleep medication	17.48%	15.19%
	Other	24.27%	29.11%

** = *p* < 0.01.

## Data Availability

The data presented in this study are available on request from the corresponding author.

## Appendix A

**Table A1:** Correlation between similar withdrawal symptoms measured with both the subjective- and objective withdrawal scale.

SWS	OWS	Pearson’s r
Craving	Craving	0.602 ***
Fatigue	Fatigue	0.488 ***
Insomnia	Insomnia	0.479 ***
Gloomy feeling	Gloomy	0.532 ***
Slow, sluggish feeling	Slow, sluggish	0.403 ***
Sweating	Sweating	0.534 ***
Tremor	Tremor/shaking hands	0.313 ***/0.409 ***
Sudden cold feeling	Sudden cold/warm feeling	0.533 ***
Muscle aches	Muscle aches	0.629 ***
Sudden warm feeling	Sudden cold/warm feeling	0.528 ***
Restless feeling	Restless	0.443 ***
Tensed, stressed feeling	Tensed, stressed	0.511 ***
Having lively dreams	Having lively dreams	0.679 ***
Eats a lot	Eats a lot	0.512 ***
Fear	Fear	0.660 ***
Yawning	Yawning	0.318 ***
Slow in movements	Slow in movements	0.403 ***
Having unpleasant dreams	Having unpleasant dreams	0.687 ***
Hungry	Hungry	0.470 ***
Muscle twitches	Muscle twitches	0.516 ***
Sleeps a lot	Sleepy/sleeps	0.399 ***
Goosebumps	Goosebumps	0.370 ***
Experience a fast heart rate	Heart rate (vital sign)	0.370 ***
Running nose	Running nose	0.461 ***
Abdominal cramps	Abdominal cramps	0.564 ***
Diarrhea	Diarrhea	0.800 ***
Tearing eyes	Tearing eyes	0.162 NS
Nausea	Nausea/Nauseous	0.479 ***/0.657 ***
Vomiting	Vomiting	0.226 **
Auditory hallucinations	Auditory hallucinations	0.826 ***
Visual hallucinations	Visual hallucinations	0.725 ***
Epileptic seizures	Epileptic seizures	−0.017 NS
Fever		
	Shivers	
	Pupil dilation	

Correlation is significant if *p* < 0.0015 (Bonferroni correction, 2-tailed). ** = *p* < 0.001; *** = *p* < 0.0001; NS = non-significant.

**Table A2:** Daily average subjective withdrawal symptoms correlated with vitals.

	Heart Rate	Systolic Blood Pressure	Diastolic Blood Pressure	N
Tapering day 1	0.164	−0.035	0.003	242
Tapering day 2	0.137	−0.078	0.038	234
Tapering day 3	0.159	−0.170	−0.023	239
Tapering day 4	0.103	−0.137	0.011	219
Tapering day 5	0.173	−0.089	−0.013	213
Tapering day 6	0.159	−0.094	0.016	203
Tapering day 7	0.127	−0.093	0.002	169
Tapering day 8	0.190	−0.006	0.051	152
Tapering day 9	0.193	−0.107	0.100	138
Tapering day 10	0.071	−0.053	0.007	116
Tapering day 11	0.167	−0.036	0.171	98

Shown values are Pearson r.

**Table A3:** Daily average objective withdrawal symptoms correlated with vitals.

	Heart Rate	Systolic Blood Pressure	Diastolic Blood Pressure	N
Tapering day 1	0.141	−0.021	0.046	263
Tapering day 2	0.133	−0.079	0.030	254
Tapering day 3	0.123	−0.005	0.086	255
Tapering day 4	0.050	−0.101	0.039	241
Tapering day 5	0.155	−0.081	0.033	233
Tapering day 6	0.055	−0.078	0.018	219
Tapering day 7	0.065	−0.091	0.037	185
Tapering day 8	0.114	0.047	0.134	168
Tapering day 9	0.172	−0.063	0.036	150
Tapering day 10	−0.040	−0.021	0.078	128
Tapering day 11	0.063	−0.043	0.157	108

Shown values are Pearson r.
